# YAP Subcellular Localization and Hippo Pathway Transcriptome Analysis in Pediatric Hepatocellular Carcinoma

**DOI:** 10.1038/srep30238

**Published:** 2016-09-08

**Authors:** Michael J. LaQuaglia, James L. Grijalva, Kaly A. Mueller, Antonio R. Perez-Atayde, Heung Bae Kim, Ghazaleh Sadri-Vakili, Khashayar Vakili

**Affiliations:** 1 Department of Surgery, Boston Children’s Hospital, Harvard Medical School, Boston, MA 02115 USA; 2 MassGeneral Institute for Neurodegenerative Disease, Massachusetts General Hospital, Harvard Medical School, Boston, MA 02129-4404 USA; 3 Department of Pathology, Boston Children’s Hospital, Harvard Medical School, Boston, MA 02115 USA

## Abstract

Pediatric hepatocellular carcinoma (HCC) is a rare tumor which is associated with an extremely high mortality rate due to lack of effective chemotherapy. Recently, the Hippo pathway and its transcriptional co-activator Yes-associated protein (YAP) have been shown to play a role in hepatocyte proliferation and development of HCC in animal models. Therefore, we sought to examine the activity of YAP and the expression of Hippo pathway components in tumor and non-neoplastic liver tissue from 7 pediatric patients with moderately differentiated HCC. None of the patients had underlying cirrhosis or viral hepatitis, which is commonly seen in adults with HCC. This highlights a major difference in the pathogenesis of HCC between children and adults. We found a statistically significant increase in YAP nuclear localization in 100% of tumors. YAP target gene (CCNE1, CTGF, Cyr61) mRNA expression was also increased in the tumors that had the most significant increase in YAP nuclear localization. Based on Ki67 co-localization studies YAP nuclear localization was not simply a marker of proliferation. Our results demonstrate a clear increase in YAP activity in moderately differentiated pediatric HCC, providing evidence that it may play an important role in tumor survival and propagation.

Hepatocellular carcinoma (HCC) is the most common type of primary liver tumor in children after hepatoblastoma^1^,^2^,^3^. A study using the Surveillance, Epidemiology and End Results (SEER) database examined a cohort of pediatric patients with HCC from 1973 to 2009, and found an overall 5-year survival of 24% and 20-year survival of 8%^4^. The HCC tumor response in the pediatric population to the commonly used regimen of cisplatin/doxorubicin (PLADO) or cisplatin/5-fluorouracil/vincristine is only about 50%^5^,^6^,^7^. Thus, patients with stage IV or metastatic disease have virtually no chance for long term survival. In contrast to hepatoblastoma, which is quite chemosensitive, no effective regimens exist for the treatment of HCC. As a result, pediatric patients with advanced HCC continue to have extremely poor long-term survival^7^,^8^.

The Hippo pathway is a tumor suppressor pathway that has gained significant attention in recent years for its role in hepatocyte proliferation and liver tumorigenesis^9^. The functional unit of this pathway, Yes-associated protein (YAP), is a transcriptional co-activator that binds and activates the transcription factor TEAD (transcriptional enhancer activator domain)^10^. Together, the YAP/TEAD complex promotes transcription of genes that stimulate proliferation and inhibit apoptosis^11^,^12^. Although YAP has been implicated in several cancers (colorectal, lung, pancreatic, and rhabdomyosarcoma^13^,^14^,^15^), its oncogenic potential was first identified and described in adult hepatocellular carcinoma (HCC)^15^,^16^. While YAP overexpression upregulates pro-survival genes, and induces HCC development in mammalian livers, decreasing YAP levels mitigate tumor formation^9^,^17^. Although a pervasive mutation within the Hippo/YAP pathway leading to HCC has not yet been identified, an increase in YAP nuclear localization has been observed in 42–85% of adult HCC tumor cells^18^,^19^,^20^. Additionally, overexpression of YAP leads to the development of HCC in mice^9^. When the Hippo pathway is active, it leads to YAP serine phosphorylation, cytoplasmic retention, and degradation^9^. Thus, YAP localizes to the nucleus only when the Hippo pathway is inactive.

The recent accumulating evidence centered on the role of the Hippo pathway in hepatocyte proliferation and adult liver tumorigenesis has implicated YAP as a potential therapeutic target for the treatment of HCC. In the adult population, HCC almost always occurs in the context of chronic liver injury and cirrhosis, commonly secondary to viral hepatitis. In contrast, outside of areas where viral hepatitis is endemic, 60–70% of HCC in the pediatric population arises in a background of non-cirrhotic liver^21^,^22^. This contrast highlights a potentially significant difference in the pathogenesis of HCC in these two populations^6^. In addition, it raises the question of whether these distinctions are also reflected in the inhibition of the Hippo pathway and YAP nuclear localization. Thus, in order to determine whether the Hippo/YAP pathway is relevant in pediatric HCC, we sought to examine the expression and subcellular localization of YAP in these tumors. We further sub-analyzed the tumors based on 2 histologic subtypes: fibrolamellar HCC (FLM-HCC), characterized by the presence of fibrous collagen bands, and non-fibrolamellar HCC (non-FLM-HCC)^23^. In addition, we examined the expression of known Hippo pathway core kinases, as well as related upstream and downstream genes.

## Results

### YAP subcellular localization and association with proliferation

**Figure 1:**
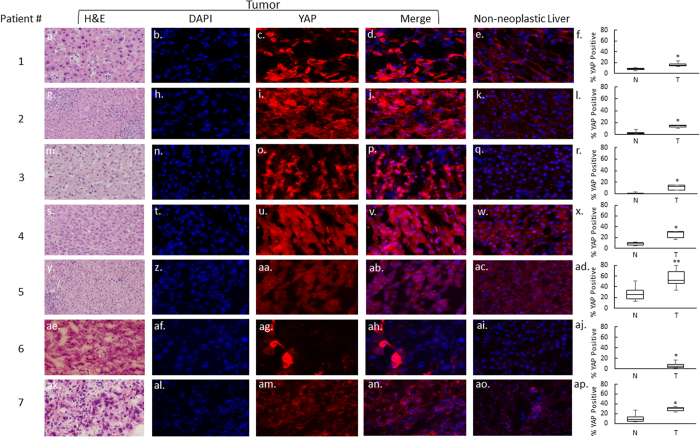
YAP nuclear localization is increased in moderately differentiated pediatric HCC compared to non-neoplastic liver. Representative sections from tumor and matched non-neoplastic liver are shown at 40x (panels a–e, g–k, m–q, s–w, y–ac, ae–ai, ak–ao). The quantitative analysis of YAP nuclear staining for each sample is shown in panels f, l, r, x, ad, aj, and ap. YAP expression in the nuclei of tumor cells is increased for all patients. *p = 0.01; **p = 0.05.

**Figure 2:**
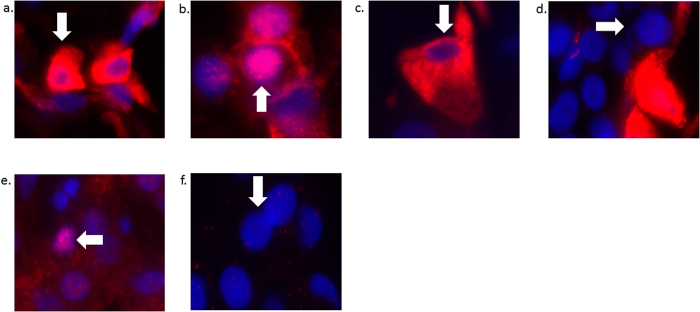
Representative images of the different staining patterns for YAP: (**a**) combined nuclear and cytoplasmic YAP staining in tumor cells (white arrow), (**b**) nuclear-only staining in tumor cells, (**c**) cytoplasmic-only staining in tumor cells, (**d**) no staining in tumor cells, (**e**) nuclear staining in non-neoplastic cells, and (**f**) no staining in non-neoplastic cells. All images were taken at 40x, then cropped and expanded to display representative cells.

**Figure 3:**
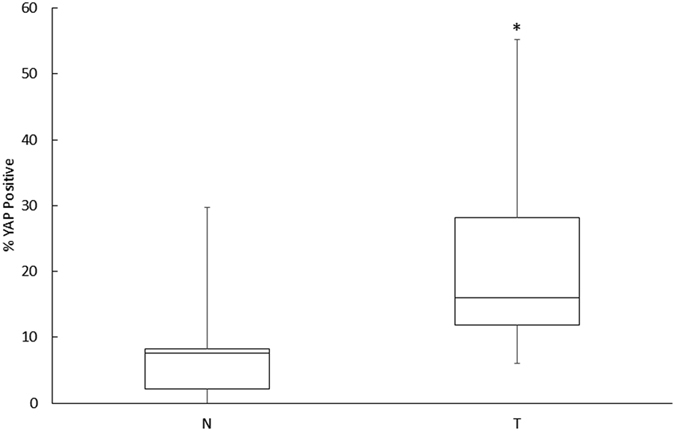
Overall levels of YAP nuclear localization in moderately differentiated pediatric HCC. The median level of YAP nuclear localization among all samples increases from 7.33% in the non-neoplastic tissue to 14.39% in the tumor tissue. *p = 0.05.

YAP is transcriptionally active when it is localized to the nucleus^11^. Therefore, we sought to determine whether YAP nuclear localization was different between tumor and non-neoplastic liver cells. All examined tumors were classified as moderately differentiated HCC by one expert liver pathologist (APA) (Table 1). YAP nuclear localization was significantly increased in 7/7 HCC tumor samples (Fig. 1). YAP subcellular localization was further subdivided into the following patterns: nuclear only, cytoplasmic only, both nuclear and cytoplasmic, or no staining (Fig. 2)^13^. Of note, all 7 tumors demonstrated both nuclear and cytoplasmic YAP. Specifically, the cytoplasmic staining seen in the tumor tissue localized immediately adjacent to the nuclei, which was a distinct pattern not seen in non-neoplastic liver. Overall, a median of 7.33% (range 0.0% to 25.47%) of non-neoplastic cells demonstrated nuclear YAP, as compared to 14.39% (range 4.6–51.7%) in the tumor samples, resulting in a significant median fold increase of 1.96 (Fig. 3).

**Figure 4:**
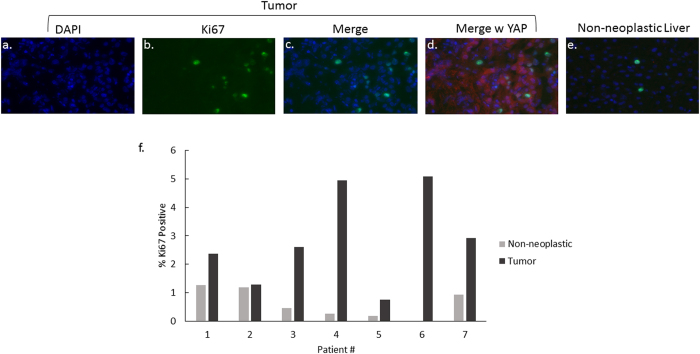
Ki67 expression in moderately differentiated pediatric HCC compared to non-neoplastic liver. Representative examples of tumor and matched non-neoplastic liver are shown at 40x (panels a–e). (Panel f) represents the percentage of Ki67 positive cells in both tumor and non-neoplastic tissue for each patient. Ki67 was increased in all 7 of the tumors.

**Figure 5:**
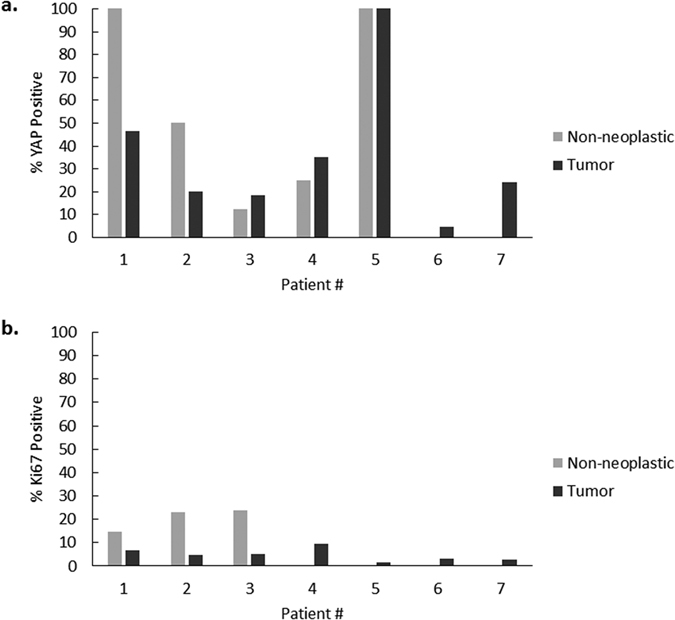
YAP and Ki67 co-localization in tumor and non-neoplastic liver. Panel a represents the percentage of Ki67 positive cells that are also YAP positive. With the exception of Patient 5, all tumors had less than 60% of Ki67 positive cells which were also YAP positive. Panel b represents the percentage of YAP positive cells that are also Ki67 positive. This constituted less than 10% of cells in all tumors.

In order to assess whether YAP nuclear localization was associated with increased cellular proliferation, the samples were co-stained for Ki67, which is expressed during the active phases of the cell cycle^24^. There was an increase in the percentage of Ki67-positive cells in all 7 tumor samples (Fig. 4), with an overall fold increase of 5.6 (0.46% to 2.6%). We subsequently examined the co-localization pattern of YAP and Ki67. The number of Ki67-positive tumor cells that also contained nuclear YAP ranged from 5% to 100%, with an overall median of 50% (Fig. 5a). Interestingly, in all tumor samples except for Patient 5, less than 60% of Ki67 positive cells were YAP positive. Patient 5 in fact had a higher percentage of YAP positive cells in both non-neoplastic and tumor cells. The number of YAP-positive tumor cells that also contained nuclear Ki67 ranged from 1.6% to 9.3%, with an overall median of 4.5% (Fig. 5b).

When the FLM-HCC group was compared to the non-FLM-HCC group, the non-FLM tumors had a slightly higher proliferative index, with 2.9% of cells demonstrating Ki67 positivity compared to 1.8% in the FLM group. This represented a 3-fold increase in the FLM group and a 7-fold increase in the non-FLM group when compared to their respective non-neoplastic livers.

For both FLM-HCC and non-FLM-HCC histology, we observed a similar increase in YAP nuclear localization and staining pattern. While the overall percentage of non-neoplastic nuclei positive for YAP was between 5% and 8% for both groups, the non-FLM-HCC had a higher overall percentage (25%) of YAP positive nuclei in the tumor cells compared to 14% in the FLM-HCC.

### YAP mRNA and protein expression

**Figure 6:**
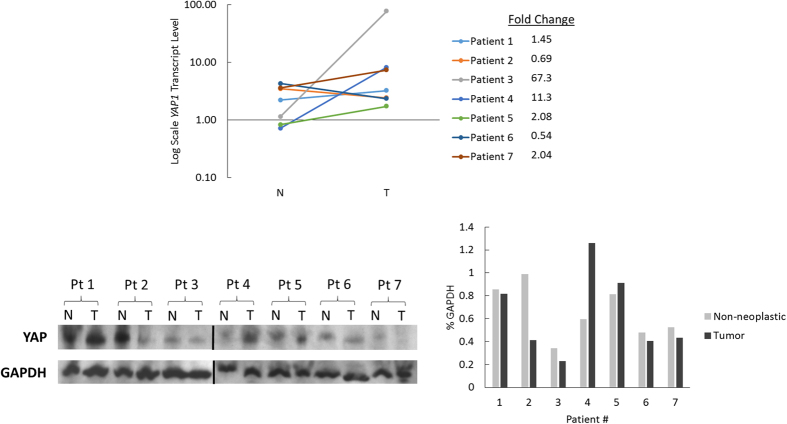
*YAP1* mRNA transcript and YAP protein levels. (**a**) Substantial increases in transcription were seen only in Patients 3, 4, and 5. The remaining patients had minimal change. **(b**) Western blot demonstrating YAP protein levels. (**c**) Bar graph quantification of immunoblot in (panel b).

Given the increased nuclear localization of YAP in tumor cells we sought to determine whether this was associated with an increase in *YAP1* transcription and protein expression. Total RNA was extracted from tumor and non-neoplastic liver samples from the same patient, and analyzed using qPCR following reverse transcription (qRT-PCR). While *YAP1* expression was upregulated by greater than 1.5 fold in Patients 3, 4, 5, and 7, there was not a significant change in *YAP1* mRNA levels between non-neoplastic liver and tumor as a cohort (Fig. 6a). When we compared FLM-HCC to non-FLM-HCC, there was a greater overall increase in *YAP1* expression in the non-FLM-HCC group; however it did not reach statistical significance. Whole cell lysate from each sample was analyzed using Western blotting (Fig. 6b,c). Only Patient 4 showed a substantial increase in YAP protein levels, correlating with the large increase this patient had in *YAP1* expression. Interestingly, although Patient 3 also had a large increase in *YAP1* expression, YAP protein levels for this patient did not change substantially. Patient 2 had a decrease in YAP protein levels, correlating with a decrease in *YAP1* expression. However, Patient 6, who also had decreased *YAP1* expression, had no substantial change in YAP protein levels. The remaining patients also had minimal change in YAP levels. There was no significant change in YAP protein expression in the cohort overall, or when the tumors were sub-analyzed based on fibrolamellar histology.

### Transcription levels of Hippo pathway components

**Figure 7:**
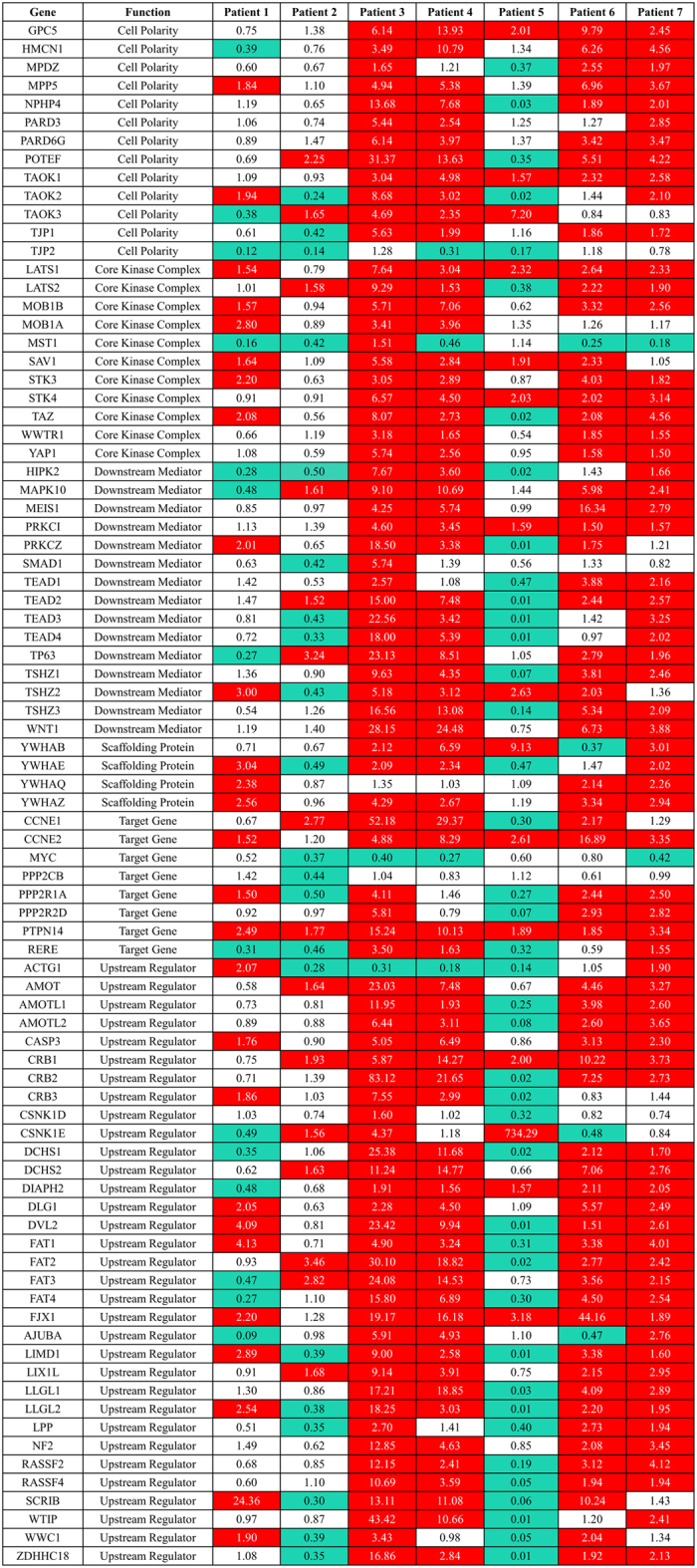
Hippo array expression heatmap. Cells highlighted in red represent genes upregulated by 50% and cells highlighted in blue represent genes downregulated by 50% or more in tumor tissue compared to non-neoplastic liver.

**Figure 8:**
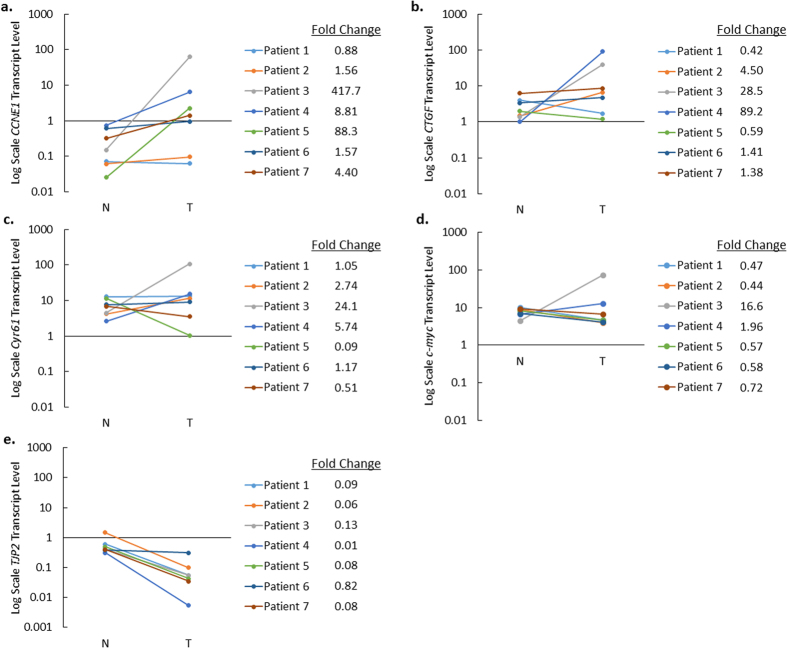
YAP target gene and Hippo pathway associated gene expression. (**a**) *CCNE1* (**b**) *CTGF* (**c**) *Cyr61* (**d**) *c-myc*. (**e**) *TJP2. CCNE1* demonstrated the most consistent increase and *TJP2* demonstrated the most consistent decrease across all tumors.

In order to assess the broader role of the Hippo pathway in the tumor samples, as well as the effect of YAP nuclear localization on known YAP target genes, we analyzed all tumor and matched non-neoplastic tissue using a commercially available PCR array containing 84 Hippo pathway-related genes (Fig. 7). There was a trend towards a statistically significant increase of YAP target genes and cell cycle regulators *CCNE1* and *CCNE2*, as well as a significant decrease in the expression of the target gene *c-myc*, a well-established oncogene^9^,^25^,^26^. There was also an upregulation of Hippo pathway components *MOB1A* and *SAV1*^27^, and cell polarization and differentiation determinants *MPP5* and *PARD6G*, which act upstream of the Hippo pathway^28^,^29^. Additionally, there was downregulation of *TJP2*, an upstream component and tumor suppressor gene, which may play a role in shuttling un-phosphorylated YAP to the nucleus^30^. To confirm the array findings, we ran individualized qRT-PCR reactions for *CCNE1*, *CCNE2*, c-*myc*, and *TJP2*, as well as previously described YAP target genes: *connective tissue growth factor (CTGF*) and *cysteine-rich angiogenic inducer 61 (Cyr61*), both of which are regulators of cell migration and proliferation^18^,^19^,^31^. All transcript levels were normalized to *HPRT1*. There was a statistically significant decrease in *TJP2* expression in tumor samples compared to non-neoplastic liver (Fig. 8e) that confirmed the findings from the Hippo arrays (p = 0.01). Although there were trends toward a significant alteration in gene expression for the other interrogated genes, *CCNE1, CTGF, Cyr61,* and *c-myc,* the findings were not statistically significant. The analysis for *CCNE1* expression for each individual sample revealed that there was a relative increase in expression in Patients 2 through 7, while Patient 1 did not show significant changes (Fig. 8a). The median fold change of *CCNE1* was higher in the non-FLM-HCC group than in the FLM-HCC group (8.8x vs. 1.5x). *CTGF* expression was increased in Patients 2, 3, 4, and demonstrated a small increase in Patients 6 and 7. However, it was decreased in Patient 1, and Patient 5 did not show any significant change in *CTGF* expression (Fig. 8b). The median fold change of *CTGF* expression was higher in the non-FLM-HCC group than in the FLM-HCC group (28.5x vs. 1.0x). There was an increase in *Cyr61* expression in Patients 2, 3, and 4, and a decrease in Patients 5 and 7. *Cyr61* expression did not change appreciably in Patients 1 or 6 (Fig. 8c). The median fold change of *Cyr61* in the non-FLM-HCC group was higher than in the FLM-HCC group (5.7x vs. 1.1x). Finally, *c-myc* was decreased in all patients in the FLM-HCC group (Patients 1, 2, 5, and 6), reaching statistical significance (p = 0.005). In the non-FLM-HCC group, there was increased *c-myc* expression in Patients 3 and 4, and decreased expression in Patient 7.

Patients with the greatest relative increase in nuclear YAP (Patients 2, 3, and 4) also demonstrated significant increases in the expression of YAP target genes *CCNE1*, *CTGF*, and *Cyr61*, with Patients 6 and 7 demonstrating only an increase in *CCNE1* expression. The remaining patients (Patients 1 and 5) had less of a relative increase in nuclear YAP. Among these patients, Patient 5 exhibited a large increase in *CCNE1* expression in tumor tissue, but the remainder of YAP target genes were either downregulated or remained unchanged. All 4 YAP target genes examined were upregulated to a greater extent in the non-FLM-HCC group compared to the FLM-HCC group.

## Discussion

Our results demonstrate that YAP nuclear localization is significantly increased in moderately differentiated pediatric HCC, however, it is not a marker of proliferation in tumor cells since on average only 50% of Ki67 positive cells were also YAP positive. In addition, we noted a distinct staining pattern in tumor cells which included significant cytoplasmic staining in addition to nuclear staining which was never observed in the non-neoplastic cells. Given the association between YAP and hepatocarcinogenesis in murine models together with the findings that YAP plays a role in hepatocyte proliferation^9^,^18^, our results suggest that the observed increase in YAP nuclear localization in pediatric HCC may be important to its biology. One of the limitations of this study is the small sample size, which is in part due to the rarity of pediatric HCC. Despite the small sample size, we were able to demonstrate that the staining pattern of YAP in HCC is quite distinct and reproducible.

The transcriptional co-activator YAP first came to light as an oncogene when genome wide studies revealed its expression was amplified in human ovarian and hepatocellular cancers, and its overexpression had transforming properties in mammary epithelial cells^16^,^32^. The oncogenic potential of YAP has been described in adult hepatocellular carcinoma (HCC)^15^,^16^ and YAP has been observed to translocate preferentially to the nucleus in 42–85% of adult HCC tumor samples^18^,^19^,^20^. Given that YAP exerts its predominant function in the nucleus, immunohistochemistry has been widely used as a method for assessing YAP activity in human cancers, with increased nuclear localization serving as a surrogate marker of YAP activity^13^,^15^,^33^. Furthermore, the induction and/or cessation of tumor growth appears to depend less on the amount of total YAP in the cell, and more on the fraction of nuclear YAP^31^. Based on previous animal studies and *in vitro* experiments, there is a high likelihood that YAP nuclear localization may be critical for the expansion of tumor cells^20^,^34^. Thus, here we have used nuclear YAP translocation as a read-out for increased YAP activity. Our results demonstrate that YAP localizes to the nucleus in 100% of moderately differentiated HCC tumors when compared to matched non-neoplastic liver tissue. These findings are comparable to previous results from adult HCC that demonstrated increased YAP nuclear staining in 42–85% of patients^18^,^19^,^20^. The majority of adult HCC arises in the background of cirrhosis, as opposed to our pediatric cohort which did not have cirrhosis. Our observation of increased nuclear YAP in pediatric tumors supports the idea that Hippo pathway suppression is not necessarily restricted to tumors arising in a background of ongoing liver injury (e.g. viral hepatitis leading to cirrhosis).

Although all of the examined tumors exhibited increased YAP staining with very similar intracellular patterns, not every tumor cell stained positive for YAP. We have previously shown that during liver regeneration in rats, YAP nuclear staining is increased during the most active phase of regeneration, with significant overlap with Ki67, a marker of proliferation^35^. Interestingly, the YAP expression pattern is different between regenerating hepatocytes following partial hepatectomy and the HCC tumor cells in our study. Importantly, the YAP expression pattern and localization in the non-neoplastic liver is also distinctly different than in tumor cells. The pattern of staining in non-neoplastic liver is most similar to that of regenerating livers, with virtually no cytoplasmic YAP staining in the non-neoplastic liver (Figs 1 and 2). This type of nuclear and cytoplasmic YAP staining has been reported in other cancers as well^13^,^15^. When we assessed the co-localization of YAP and Ki67 in tumor samples, we noted that most Ki67-positive cells did not contain YAP in the nucleus. Overall, only 50% of Ki67 positive cells were YAP positive in tumor samples and an overwhelming majority of YAP positive cells (nuclear and cytoplasmic) were not Ki67 positive. These findings raise two possibilities: 1) YAP activity precedes tumor cell entry into the active phase of cell cycle, or 2) YAP activity in some tumor cells may have non-autonomous activity resulting in proliferation of adjacent tumor cells. Considering the co-localization pattern with Ki67, as well as the pattern of YAP staining in HCC cells and non-neoplastic cells, we hypothesize that the Hippo/YAP pathway may function differently in tumor cells than in normal or regenerating hepatocytes. This is supported by the fact that only a small fraction of YAP-positive cells are also Ki67-positive, indicating that YAP may play a role in these tumors outside of active cellular proliferation, such as preventing apoptosis or maintaining a de-differentiated stem-like state. This is in contrast to our observations in normal regenerating livers, where nearly 100% of YAP-positive cells are Ki67-positive. Furthermore, the localization of YAP to both the nuclei and immediately adjacent cytoplasm of tumor cells is a unique pattern, and is not observed in either non-neoplastic cells from the same patient cohort, or in normal regenerating liver cells that contain nuclear YAP. Whether the cytoplasmic YAP represents a pool of protein in the process of shuttling to the nucleus or serves an alternate function in the cytoplasm is unclear. The interaction of cytoplasmic phosphorylated YAP with other signaling pathways (e.g. Wnt, NOTCH, beta-TGF) has been previously described^18^,^36^,^37^,^38^,^39^. Therefore, this interaction may contribute significantly to the virulence of the tumor cell, and suggests that possibly both nuclear and cytoplasmic YAP may contribute to the phenotype of the tumor.

Even though our immunofluorescence analysis demonstrated significant increases in YAP staining in tumor cells, this was not associated with an overall increase in *YAP1* transcription or protein expression. This finding is similar to previous results and suggests that nuclear localization depends less on increased YAP expression, and more on YAP dephosphorylation, resulting in decreased protein degradation and increased trafficking into the nucleus^16^,^31^. We attempted to quantify phosphorylated YAP levels in the tumor samples using immunofluorescence and immunoblots, however, our findings were confounded by the lack of availability of reliable antibodies for human tissue.

YAP is a co-activator of transcription, binding to the TEAD complex in the nucleus and allowing TEAD to directly activate its target genes. Numerous target genes that promote cell proliferation and inhibit differentiation have been associated with YAP; however, they demonstrate variable activity depending on tissue type^9^,^13^,^40^. Previous studies have shown that YAP target genes such as *BIRC5*, *glypican 3*, *alpha fetoprotein (AFP*), and *CTGF* are elevated in both murine and adult human HCC samples^18^,^19^,^31^. Furthermore, *CTGF* and *Cyr61* expression were shown to be dependent on YAP expression in hepatoblastoma cells^18^. However, the expression level of these genes was not previously quantified in pediatric HCC. Using a commercial array followed by confirmatory qRT-PCR analysis, we found an overall increase in *CCNE1, CTGF,* and *Cyr61* expression in tumor samples compared to control when grouping all HCC samples. However, there was a significant difference in gene expression pattern between individual tumors. Interestingly, tumors with significant increases in YAP nuclear localization (Patients 2, 3, and 4) also demonstrated significant upregulation of target genes, suggesting an increase in YAP transcriptional activity. While Patients 5, 6, and 7 demonstrated a significant increase in nuclear YAP in tumor compared to non-neoplastic tissue, they only had a substantial increase in *CCNE1* expression, with *Cyr61* being downregulated in Patient 5 and unchanged in Patients 6 and 7, and *CTGF* unchanged in all three patients. Patient 1 demonstrated a significant increase in nuclear YAP in tumor compared to non-neoplastic tissue, however it was increased by a smaller fraction compared to the other patients. This patient exhibited a decrease in *CTGF*, no change in the expression of other target genes, and had fewer proliferating cells in the tumor sample compared to most of the other patients. This variability in gene expression patterns of “known” YAP target genes may be due to not only tissue-specificity, but also tumor specificity, depending on the driver mutations and other interacting signaling pathways. Future studies focusing on YAP/TEAD interactions using chromatin immunoprecipitation, followed by subsequent sequence analysis, will be necessary to identify tumor-specific target genes.

**Figure 9:**
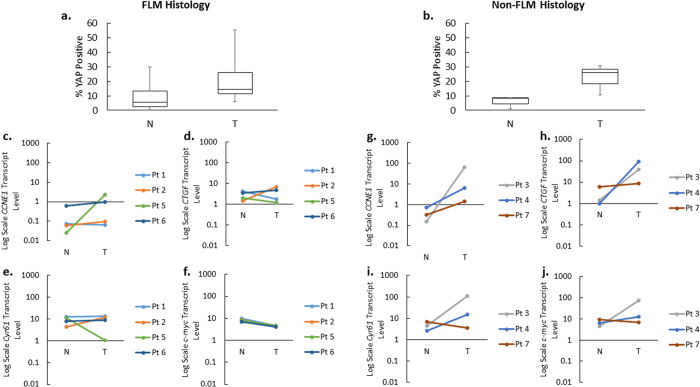
YAP localization and gene expression based on fibrolamellar histology. The median level of YAP nuclear localization increased from 5.4% to 14.6% in FLM-HCC (**a**) and from 8.1% to 25.8% in non-FLM-HCC (**b**). Non-FLM-HCC also had a greater upregulation of YAP target genes *CCNE1* (**c,g**), *CTGF* (**d,h**), *Cyr61* (**e,i**), and *c-myc* (**f,j**).

FLM-HCC is a subtype of HCC characterized by fibrous collagen bands on histology, that tends to affect the pediatric and young adult population, without a background of cirrhosis^23^. It has recently been linked to a deletion on chromosome 19, leading to the chimeric fusion protein DNAJB1-PRKACA, which has been proposed as a driver mechanism, though confirmatory evidence is still being collected^41^. This fusion event has a reported frequency of 79–100% in FLM-HCC^41^,^42^. This high concordance between the histologic findings and the potential genetic driver suggests that FLM-HCC may demonstrate a uniform form of tumor compared to non-FLM-HCC. We therefore sub-analyzed the tumors based on fibrolamellar histology. In our cohort, 4/7 tumors (Patients 1, 2, 5, and 6) were FLM-HCC. Overall, non-FLM-HCC tumors had a greater degree of YAP nuclear localization, proliferative index, and upregulation of the YAP target genes *CCNE1, CTGF,* and *Cyr61* compared to FLM-HCC (Fig. 9). Interestingly, FLM-HCC also had a significant downregulation of *c-myc,* although the mechanistic implications of this may be of interest in future studies. Clinically, FLM-HCC is known to have a slightly better prognosis than non-FLM-HCC. Whether greater YAP nuclear activity in non-FLM-HCC may be a contributing factor remains to be elucidated.

One of our most consistent findings was the decrease in *TJP2* transcript levels. *TJP2* is a tight junction scaffold protein involved in cell contact-mediated growth inhibition, preventing entry into the cell cycle^43^. It has previously been implicated as a tumor suppressor gene in both lung and pancreatic cancers^44^,^45^. In cell lines, *TJP2* has been observed to play a direct role in shuttling unphosphorylated YAP to the nucleus^30^,^46^. Our observation of a statistically significant reduction in *TJP2* mRNA expression in every patient in our cohort appears to be most consistent with its suspected role as a tumor suppressor gene.

YAP nuclear localization is not observed in all tumor cells, highlighting a heterogeneous population of cells. Therefore, the use of whole-cell extracts from the tumors does not restrict the analysis to only the YAP positive cells and may result in the “dilution” of the RNA expression results. Indeed this is a limitation of the current study in which whole-cell tumor extracts were used for the analysis of a subpopulation of cells. However, as discussed above, it is still critical to highlight the findings that in tumor samples with the greatest increase in YAP staining there was a positive correlation with alterations in *CCNE1*, *Cyr61*, and *CTGF* expression. Studies aimed at comparing the gene expression profiles of YAP positive and YAP negative cell populations may shed light onto the differences between these two distinct populations of cells.

## Materials and Methods

### Tumor Samples

Seven hepatocellular carcinoma samples, with matched controls from non-neoplastic liver tissue within the same lobe, but far from the tumor, were obtained from the excised livers of patients undergoing liver transplantation or hepatic resection. All were classified as being moderately differentiated by staff pathologists based on mitotic index and cytologic grade, using a modification of the Edmonson-Steiner grading system (Table 1)^47^. Tumor tissue and non-neoplastic tissue from the same resected specimen were identified and sampled under the guidance of staff pathologists. The study protocol was approved by the Institutional Review Board at Boston Children’s Hospital, and carried out in accordance with their guidelines. Written and informed consent was obtained from the parents or guardians of all subjects.

### Immunostaining

Fresh frozen liver tissue was sectioned at 8 μm and stained using previously described methods^35^. Anti-YAP antibody and anti-Ki67 antibody (Cell Signaling, Beverly, MA) were used for primary incubation. For secondary antibody incubation we utilized Cy3-conjugated goat anti-rabbit and FITC-conjugated goat anti-mouse antibodies (Jackson ImmunoResearch, West Grove, PA). The data was analyzed using open source software from the National Institutes of Health (ImageJ).

### Hematoxylin and Eosin staining

Sections of fresh frozen liver tissue were sectioned at 8 μm and dehydrated in absolute methanol for 1 min. The slides were stained in Harris’ Hematoxylin (Sigma Aldrich, St. Louis, MO) for 2 min, followed by a 20 second wash in water. The pH was then reduced with a 1 minute wash in bluing reagent (Fisher Scientific, Cambridge, MA). The slides were washed for 30 seconds in water, followed by 30 seconds in 95% ethanol, and then stained in Eosin Yellow (Fisher Scientific) for 1 minute. The sections were then dehydrated in increasing concentrations of ethanol, followed by xylenes. Finally, the slides were mounted in Permount toluene solution (Fisher Scientific) and imaged using light microscopy.

### Image Analysis

Five 20x fields were photographed from one section of tumor and non-neoplastic tissue. At least 1500 cells were examined per sample. For each field, the total number of nuclei staining positive for DAPI were counted using ImageJ software. The number of nuclei staining positive for YAP against background were then counted manually and expressed as a percentage of the total nuclei. This process was repeated for nuclei staining positive for Ki67, as well as nuclei staining positive for both YAP and Ki67. A median ± interquartile range was generated from all samples. The Mann-Whitney-U test for non-parametric distributions was used for comparisons between non-neoplastic (control) livers and tumor tissue. Each tumor sample was compared to non-neoplastic liver from the same patient. YAP nuclear staining was considered to be increased or decreased in tumor samples if the interquartile range did not overlap with that of the control. In addition, the overall pattern of YAP staining was categorized as: 1) nuclear only, 2) cytoplasmic only, 3) both nuclear and cytoplasmic, or 4) no staining (Fig. 2). P-value less than or equal to 0.05 was considered statistically significant.

### RNA extraction and reverse transcription

RNA was extracted from snap-frozen liver tissue using an RNeasy kit (Qiagen, Valencia, CA) according to the manufacturer’s instructions and as previously described^35^. Reverse transcription reactions were performed using Superscript First Strand Synthesis System (Invitrogen, Carlsbad, CA) in an iCycler (Bio-Rad, Berkeley, CA).

### Hippo Pathway Arrays and Quantitative Real Time PCR

The RT2 Profiler PCR Array for Human Hippo Signaling Pathway, with SYBR-green PCR Master Mix (Qiagen, Hilden, Germany) was used to assess gene expression. The array contains primers for 84 different Hippo pathway-related genes (See Fig. 7 for list of genes). The threshold cycle for each sample was chosen from the linear range and converted to a starting quantity by interpolation from a standard curve run on the same plate for each set of primers. For each replicate, mRNA levels were normalized to their respective *HPRT1* mRNA levels. The transcript levels are expressed as 2^−ΔCT^, where the ΔCT is the difference between the cycle thresholds of the gene of interest and *HPRT1* for each sample. Confirmatory PCR was subsequently performed in duplicate, with the crossing threshold for each well within 0.2 cycles of its counterpart. Statistical comparisons were made using the Student’s t-test. The following primers were used: *Yes-associated protein (YAP1*): forward 5′-TGAACAAACGTCCAGCAAGATAC-3′ and reverse 5′-CAGCCCCCAAAATGAACAGTAG-3′; *cyclin E1 (CCNE1)*: forward 5′-AAGGAGCGGGACACCATGA-3′ and reverse 5′-ACGGTCACGTTTGCCTTCC-3′; *cyclin E2 (CCNE2)*: forward 5′-GCCGAGCGGTAGCTGGTC-3′ and reverse 5′-GGGCTGCTGCTTAGCTTGTAAA-3′; *avian myelocytomatosis viral oncogene homologue (c-myc)*: forward 5′-GTCAAGAGGCGAACACACAAC-3′ and reverse 5′-TTGGACGGACAGGATGTATGC-3′; *tight junction protein 2 (TJP2*): forward 5′-GCGAGAAGCTGGTTTCAAGAGA-3′ and reverse 5′-GCTTTCGGAGTCACATCCAGTA-3′, and *hypoxanthine phosphoribosyltransferase 1* (HPRT1): forward 5′-TGAGGATTTGGAAAGGGTGT-3′ and reverse 5′-GAGCACACAGAGGGCTACAA-3′ in an iCycler. Further real time PCR studies were performed on previously described^18^,^19^,^31^ YAP target genes *connective tissue growth factor (CTGF*): forward 5′-GCCACAAGCTGTCCAGTCTAATCG-3′ and reverse 5′-TGCATTCTCCAGCCATCAAGAGAC-3′; and *cysteine*-*rich angiogenic protein 61 (Cyr61*): forward 5′-TTCTTTCACAAGGCGGCACTC-3′ and reverse 5′-AGCCTCGCATCCTATACAACC-3′.

### Western Blot Analysis

Western blots were carried out as previously described^48^,^49^. Briefly, 50–100 μg of protein from whole cell extracts from both tumor and non-neoplastic liver were re-suspended in 4X tris-glycine sample buffer, boiled at 95 °C for 5–10 min, and fractionated on a 4–20% tris-glycine gel (Invitrogen, Carlsbad, CA) for 90 min at 125–130 V. Proteins were transferred to PVDF membranes and then blocked with a solution of either 5% BSA or 3% BSA and 5% milk in tris-buffered saline with Tween 20 (TBST) before immunodetection with the following antibodies: anti-YAP (Cell Signaling #4912, Danvers, MA), and anti-GAPDH (Millipore #MAB374, Billerica, MA). Overnight primary antibody incubation was followed by washes (60 min at room temperature) in TBST before incubation in secondary antibody for 1 hour (horseradish peroxidase-conjugated goat anti-rabbit IgG, Cell Signaling; or goat anti-mouse IgG, Bio-Rad, Hercules, CA). After washing with TBST, proteins were visualized using the ECL detection system (NEN, Boston, MA). Coomassie gels were used to ensure equal protein loading. Band densities were analyzed using AlphaEase FC software version 4.1.0 (Alpha Innotech) to determine relative protein expression, and were then normalized to GAPDH band densities.

## Additional Information

**How to cite this article**: LaQuaglia, M. J. *et al.* YAP Subcellular Localization and Hippo Pathway Transcriptome Analysis in Pediatric Hepatocellular Carcinoma. *Sci. Rep.* **6**, 30238; doi: 10.1038/srep30238 (2016).

## Figures and Tables

**Table 1:** Patient and tumor characteristics.

Patient #	Age (years)	Gender	Surgery	Neoadjuvant chemotherapy	Tumor pathology	Histologic vascular invasion
1	13	M	Hepatic resection	Y	Fibrolamellar type HCC, moderately differentiated	Y
2	13	M	Multivisceral transplant	Y	Fibrolamellar type HCC, moderately differentiated	Y
3	1.5	M	Hepatic resection	Y	HCC, moderately differentiated	Y
4	13	M	Hepatic resection	Y	HCC, moderately differentiated	Y
5	11	M	Hepatic resection	Y	Fibrolamellar type HCC, moderately differentiated	Y
6	15.5	M	Hepatic resection	Y	Fibrolamellar type HCC, moderately differentiated	Y
7	10	M	Hepatic resection	Y	HCC, moderately differentiated	Y
